# Algorithms for the common solution of the split variational inequality problems and fixed point problems with applications

**DOI:** 10.1186/s13660-018-1942-1

**Published:** 2018-12-29

**Authors:** Panisa Lohawech, Anchalee Kaewcharoen, Ali Farajzadeh

**Affiliations:** 1 Department of Mathematics, Faculty of Science, Naresuan University, Phitsanulok, Thailand; 2 Department of Mathematics, Faculty of Science, Razi University, Kermanshah, Iran

**Keywords:** 58E35, 47H10, Variational inequality problems, Extragradient methods, CQ algorithms

## Abstract

In this paper, we establish a new iterative algorithm by combining Nadezhkina and Takahashi’s modified extragradient method and Xu’s algorithm. The mentioned iterative algorithm presents the common solution of the split variational inequality problems and fixed point problems. We show that the sequence produced by our algorithm is weakly convergent. Finally, we give some applications of the main results. This article extends the previous results in this area.

## Introduction

Variational inequality problem (VIP) is the problem of finding a point $x^{*}$ in a subset *C* of a Hilbert space *H* such that

1.1 $$ \bigl\langle f\bigl(x^{*}\bigr), x - x^{*} \bigr\rangle \geq0\quad \text{for all } x \in C, $$

where $f:C \rightarrow H$ is a mapping, and we denote its solution set of (1.1) by $\operatorname{VI}(C,f)$. The VIP was introduced by Stampacchia [24]. In 1966, Hartman and Stampacchia [17] suggested the VIP as a tool for the study of partial differential equations. The ideas of the VIP are being applied in many fields including mechanics, nonlinear programming, game theory, economics equilibrium, and so on. Moreover, it contains fixed point problems, optimization problems, complementarity problems, and systems of nonlinear equations as special cases (see [3, 12, 20–22, 29, 38, 40, 41]). Using the projection technique in [26], we know that the VIP is equivalent to the fixed point problem, that is,

$$ x^{*} \in\operatorname{VI}(C,f) \quad \text{if and only if}\quad x^{*} = P_{C}(I - \gamma f)x^{*}, $$

where $\gamma> 0$ and $P_{C}$ is the metric projection of *H* onto *C*. In [36], the following sequence $\{x_{n}\}$ of Picard iterates is a strongly convergent sequence in $\operatorname{VI}(C,f)$ because $P_{C}(I - \gamma f)$ is a contraction on *C*, where *f* is *η*-strongly monotone and *k*-Lipschitz continuous, $0 < \gamma< \frac{2\eta}{k^{2}}$:

1.2 $$ x_{n+1} = P_{C}(I - \gamma f)x_{n}. $$

However, algorithm (1.2) cannot be used to solve VIP when *f* is monotone and *k*-Lipschitz continuous, which can be seen from the counterexample in [43]. During the last decade, many authors devoted their attention to studying algorithms for solving the VIP. One of the methods is the extragradient method which was introduced and studied in 1976 by Korpelevich [19] in the finite dimensional Euclidean space ${\mathbb {R}}^{n}$:

1.3 $$ \begin{aligned} &y_{n} = P_{C}(x_{n} - \gamma fx_{n}), \\ &x_{n+1} = P_{C}(x_{n} - \gamma fy_{n}), \end{aligned} $$

when *f* is monotone and *k*-Lipschitz continuous. Then sequence $\{ x_{n}\}$ converges to the solution of VIP.

Takahashi and Toyoda [28] illustrated that if $S:C \rightarrow C$ is a nonexpansive mapping and *I* is the identity mapping on *H*, then $f=I-S$ is $\frac{1}{2}$-inverse strongly monotone and $\operatorname{VI}(C,f)= \operatorname{Fix}(S)$. Motivated and inspired by the mentioned fact, they introduced and studied the following method for finding a common element of $\operatorname{VI}(C,f)\cap\operatorname{Fix}(S)$:

1.4 $$ x_{n+1}=\lambda_{n} x_{n}+(1-\lambda_{n})SP_{C}(x_{n}- \gamma_{n} fx_{n}), $$

when $S: C \rightarrow C$ is a nonexpansive mapping and $f:C \rightarrow H$ is a *ν*-inverse strongly monotone mapping.

After that, Nadezhkina and Takahashi [27] suggested the following modified extragradient method motivated by the idea of Korpelevich [19]:

1.5 $$ \begin{aligned} &y_{n} = P_{C}(x_{n}- \gamma_{n} fx_{n}), \\ &x_{n+1} = \lambda_{n} x_{n}+(1- \lambda_{n})SP_{C}(x_{n}-\gamma_{n} fy_{n}), \end{aligned} $$

when $S: C \rightarrow C$ is a nonexpansive mapping and $f:C \rightarrow H$ is a monotone and *k*-Lipschitz continuous mapping. They showed that the sequence generated by the mentioned method converges weakly to an element in $\operatorname{VI}(C,f)\cap\operatorname{Fix}(S)$.

Since then, it has been used to study the problems of finding a common solution of VIP and fixed point problem (see [42] and the references therein).

The split feasibility problem (SFP) proposed by Censor and Elfving [10] is finding a point

1.6 $$ x \in C \quad \text{and} \quad Ax \in Q, $$

where *C* and *Q* are nonempty closed convex subsets of real Hilbert spaces $H_{1}$ and $H_{2}$, respectively, and $A:H_{1} \rightarrow H_{2}$ is a bounded linear operator. Since then, the SFP has been widely used in many applications such as signal processing, intensity-modulation therapy treatment planning, phase retrievals and other fields (see [5, 6, 9, 15, 18, 37] and the references therein).

One of the popular methods for solving the SFP is the CQ algorithm presented by Byrne [5] in 2002 as follows:

1.7 $$ x_{n+1}= P_{C}\bigl(x_{n} - \gamma A^{*}(I-P_{Q})Ax_{n}\bigr), $$

where $0 < \gamma< \frac{2}{\|A\|^{2}}$ and $A^{*}$ is the adjoint operator of *A*.

Since (1.7) can be viewed as a fixed point algorithm for averaged mappings, Xu [34] applied the K-M algorithm to present the following algorithm for solving the SFP:

1.8 $$ x_{n+1}=(1-\alpha_{n})x_{n}+\alpha_{n} P_{C}\bigl(x_{n} - \gamma A^{*}(I-P_{Q})Ax_{n} \bigr). $$

The split variational inequality problem (SVIP) is the problem of finding a point

1.9 $$ \begin{aligned} &x^{*} \in C \quad \text{such that}\quad \bigl\langle f\bigl(x^{*}\bigr), x - x^{*} \bigr\rangle \geq 0,\quad \text{for all } x \in C,\quad \text{and} \\ &y^{*}=Ax^{*} \in Q \quad \text{solves}\quad \bigl\langle g\bigl(y^{*}\bigr), y - y^{*} \bigr\rangle \geq 0, \quad \text{for all } y \in Q, \end{aligned} $$

where *C* and *Q* are nonempty closed convex subsets of real Hilbert spaces $H_{1}$ and $H_{2}$, respectively, $f:H_{1} \rightarrow H_{1}$ and $g:H_{2} \rightarrow H_{2}$ are mappings, and $A: H_{1} \rightarrow H_{2}$ is a bounded linear operator. The SVIP was first investigated by Censor et al. [11]; it includes split feasibility problem, split zero problem, variational inequality problem and split minimizations problem as special cases (see [5, 7, 11, 16, 31, 39]).

In 2017, Tian and Jiang [32] considered the following iteration method by combining extragradient method with CQ algorithm for solving the SVIP:

1.10 $$\begin{aligned}& y_{n} = P_{C}\bigl(x_{n} - \gamma_{n} A^{*}\bigl(I - P_{Q}(I-\theta g)\bigr)Ax_{n}\bigr), \\& z_{n} = P_{C}\bigl(y_{n} - \lambda_{n}f(y_{n}) \bigr), \\& x_{n+1} = P_{C}\bigl(y_{n} - \lambda_{n}f(z_{n}) \bigr), \end{aligned}$$

where $A:H_{1} \rightarrow H_{2}$ is a bounded linear operator, $f:C \rightarrow H_{1}$ is a monotone and *k*-Lipschitz continuous mapping, and $g:H_{2} \rightarrow H_{2}$ is a *δ*-inverse strongly monotone mapping.

In this paper, we establish a new iterative algorithm by combining Nadezhkina and Takahashi’s modified extragradient method and Xu’s algorithm. The mentioned iterative algorithm presents the common solution of the split variational inequality problems and fixed point problems. We show that the sequence produced by our algorithm is weakly convergent. Finally, we give some applications of the main results. This article extends the results that appeared in [32].

## Preliminaries

In order to solve the our results, we recall the following definitions and preliminary results that will be used in the sequel. Throughout this section, let *C* be a closed convex subset of a real Hilbert space *H*.

A mapping $T:C \rightarrow C$ is said to be *k*-Lipschitz continuous with $k>0$, if

$$ \|Tx-Ty\| \leq k\|x-y\| $$

for all $x, y \in C$. A mapping *T* is said to be nonexpansive when $k=1$. We say that $x \in C$ is a fixed point of *T* if $Tx=x$ and the set of all fixed points of *T* is denoted by $\operatorname{Fix}(T)$. It is well known that if *C* is a nonempty bounded closed convex subset of *H* and $T:C \rightarrow C$ is nonexpansive, then $\operatorname{Fix}(T) \neq\emptyset$. Moreover, for a fixed $\alpha\in(0,1)$, a mapping $T:H \rightarrow H$ is *α*-averaged if and only if it can be written as the average of the identity mapping on *H* and a nonexpansive mapping $S:H \rightarrow H$, i.e.,

$$ T = (1-\alpha)I+\alpha S. $$

Recall that a mapping $f: C \rightarrow H$ is called *η*-strongly monotone with $\eta> 0$ if

$$ \langle fx-fy, x-y \rangle\geq\eta\|x-y\|^{2} $$

for all $x, y \in C$. If $\eta=0$, then the mapping *f* is said to be monotone. Further, a mapping *f* is said to be *ν*-inverse strongly monotone with $\nu>0$ (*ν*-ism) if

$$ \langle fx-fy, x-y \rangle\geq\nu\|fx-fy\|^{2} $$

for all $x, y \in C$. In [1], we know that a *η*-strongly monotone mapping *f* is monotone and a *ν*-ism mapping *f* is monotone and $\frac{1}{\nu}$-Lipschitz continuous. Moreover, $I-\lambda f$ is nonexpansive where $\lambda\in(0,2\nu)$, see [34] for more details on averaged and *ν*-ism mappings.

### Lemma 2.1

([8])

*Given* $x \in H$ *and* $z \in C$. *Then the following statements are equivalent*:

(i) $z = P_{C}x$;
(ii) $\langle x - z, z - y \rangle\geq0$ *for all* $y \in C$;
(iii) $\|x - y\|^{2} \geq\|x - z\|^{2} + \|y - z\|^{2}$ *for all* $y \in C$.

We need the following definitions about set-valued mappings for proving our main results.

### Definition 2.2

([30])

Let $B:H \rightrightarrows H$ be a set-valued mapping with the effective domain $D(B) = \{x \in H : Bx \neq\emptyset\}$.

The set-valued mapping *B* is said to be monotone if, for each $x, y \in D(B)$, $u \in Bx$, and $v \in By$, we have

$$\langle x - y,u - v\rangle\geq0. $$

Also the monotone set-valued mapping *B* is said to be maximal if its graph $G(B)=\{(x, y) : y \in Bx\}$ is not properly contained in the graph of any other monotone set-valued mappings.

The following property of the maximal monotone mappings is very convenient and helpful to use:

A monotone mapping *B* is maximal if and only if, for $(x,u) \in H \times H$,

$$\langle x - y,u - v \rangle\geq0 \quad \text{for each } (y, v) \in G(B) \quad \text{implies}\quad u \in Bx. $$

For a maximal monotone set-valued mapping *B* on *H* and $r > 0$, the operator

$$J_{r} := (I + rB)^{-1} : H \rightarrow D(B) $$

is called the resolvent of *B*.

### Remark 2.3

In [14], we obtain that $\operatorname{Fix}(J_{r}) = B^{-1}0$ for all $r > 0$ and $J_{r}$ is firmly nonexpansive, that is,

$$\|J_{r}x - J_{r}y\|^{2} \leq\langle J_{r}x-J_{r}y,x-y \rangle\quad \text{for all } x,y \in H. $$

Indeed, by the definition of scalar multiplication, addition, and inversion operations, we have

$$(x,y)\in G(B) \quad \Leftrightarrow\quad (x+ry,x)\in(I+rB)^{-1}=J_{r}. $$

Hence, for all $(x,y),(x^{*},y^{*})\in G(B)$, we get

$$\begin{aligned} B \text{ is monotone} \quad \Leftrightarrow\quad & \bigl\langle x^{*}-x, y^{*} - y \bigr\rangle \geq0 \\ \quad \Leftrightarrow\quad & \bigl\langle x^{*}-x, ry^{*} - ry \bigr\rangle \geq0 \\ \quad \Leftrightarrow\quad & \bigl\langle x^{*}-x, x^{*}-x+ry^{*} - ry \bigr\rangle \geq \bigl\Vert x^{*}-x \bigr\Vert ^{2} \\ \quad \Leftrightarrow\quad & \bigl\langle J_{r}\bigl(x^{*}+ry^{*} \bigr)-J_{r}(x+ry), \bigl(x^{*}+ry^{*}\bigr) - (x+ry)\bigr\rangle \\ &\quad \geq \bigl\Vert J_{r}\bigl(x^{*}+ry^{*}\bigr)-J_{r}(x+ry) \bigr\Vert ^{2} \\ \quad \Leftrightarrow\quad & J_{r} \text{ is firmly nonexpansive}. \end{aligned}$$

Let $f:C \rightarrow H$ be a monotone and *k*-Lipschitz continuous mapping. In [2], we know that a normal cone to *C* defined by

$$N_{C}x = \bigl\{ z \in H : \langle z, y - x \rangle\leq0, \text{for all } y \in C\bigr\} \quad \text{for all } x \in C $$

is a maximal monotone mapping and a resolvent of $N_{C}$ is $P_{C}$.

The following results play the crucial role in the next section.

### Lemma 2.4

([27])

*Let* $H_{1}$ *and* $H_{2}$ *be real Hilbert spaces*. *Let* $B : H_{1} \rightrightarrows{H_{1}}$ *be a maximal monotone mapping and* $J_{r}$ *be the resolvent of* *B* *for* $r > 0$. *Suppose that* $T : H_{2} \rightarrow H_{2}$ *is a nonexpansive mapping and* $A : H_{1} \rightarrow H_{2}$ *is a bounded linear operator*. *Assume that* $B^{-1}0 \cap A^{-1}\operatorname{Fix}(T) \neq\emptyset$. *Let* $r, \gamma> 0 $ *and* $z \in H_{1}$. *Then the following statements are equivalent*:

(i) $z = J_{r}(I - \gamma A^{*}(I - T)A)z$;
(ii) $0 \in A^{*}(I - T)Az + Bz$;
(iii) $z \in B^{-1}0 \cap A^{-1}\operatorname{Fix}(T)$.

### Lemma 2.5

([23])

*Let* $\{\alpha_{n}\}$ *be a real sequence satisfying* $0< a \leq\alpha _{n}\leq b< l$ *for all* $n \geq0$, *and let* $\{v_{n}\}$ *and* $\{w_{n}\}$ *be two sequences in* *H* *such that*, *for some* $\sigma\geq0$,

$$\begin{aligned}& \limsup_{n \rightarrow \infty}\|v_{n}\| \leq \sigma, \\& \limsup_{n \rightarrow \infty}\|w_{n}\| \leq \sigma, \\& \textit{and}\quad \lim_{n \rightarrow \infty} \bigl\Vert \alpha_{n}v_{n}+(1- \alpha_{n})w_{n} \bigr\Vert =\sigma. \end{aligned}$$

*Then* $\lim_{n \rightarrow \infty}\|v_{n}-w_{n}\|=0$.

### Lemma 2.6

([35])

*Let* $\{x_{n}\}$ *be a sequence in* *H* *satisfying the properties*:

(i) $\lim_{n \rightarrow \infty}\|x_{n}-u\|$ *exists for each* $u \in C$;
(ii) $\omega_{w}(x_{n}) \subset C$.

*Then* $\{x_{n}\}$ *converges weakly to a point in* *C*.

### Theorem 2.7

([27])

*Let* $f:C \rightarrow H$ *be a monotone and* *k*-*Lipschitz continuous mapping*. *Assume that* $S:C \rightarrow C$ *is a nonexpansive mapping such that* $\operatorname {VI}(C,f)\cap\operatorname{Fix}(S) \neq\emptyset$. *Let* $\{x_{n}\}$ *and* $\{ y_{n}\}$ *be sequences generated by* (1.5), *where* $\{\lambda_{n}\}\subset[a,b]$ *for some* $a,b \in(0,\frac{1}{k})$ *and* $\{\alpha_{n}\} \subset[c,d]$ *for some* $c,d \in(0,1)$. *Then the sequences* $\{x_{n}\}$ *and* $\{y_{n}\}$ *converge weakly to the same point* $z \in\operatorname{VI}(C,f)\cap\operatorname{Fix}(S) \neq\emptyset$, *where* $z = \lim_{n \rightarrow \infty} P_{\operatorname{VI}(C,f)\cap\operatorname{Fix}(S)}x_{n}$.

### Theorem 2.8

([34])

*Assume that the solution set of SFP is consistent and* $0< \gamma< \frac{2}{\|A\|^{2}}$. *Let* $\{x_{n}\}$ *be defined by the averaged CQ algorithm* (1.8) *where* $\{\alpha_{n}\}$ *is a sequence in* $[0,\frac{4}{2+\gamma\|A\|^{2}} ]$ *satisfying the condition*

$$\sum_{n=1}^{\infty} \alpha_{n} \biggl( \frac{4}{2+\gamma\|A\| ^{2}}-\alpha_{n} \biggr)= \infty. $$

*Then the sequence* $\{x_{n}\}$ *is weakly convergent to a point in the solution set of SFP*.

## Main results

Our aim in this section is to consider an iterative method by combining Nadezhkina and Takahashi’s modified extragradient method with Zhao and Yang’s algorithm for solving the split variational inequality problems and fixed point problems.

Throughout our results, unless otherwise stated, we assume that *C* and *Q* are nonempty closed convex subsets of real Hilbert spaces $H_{1}$ and $H_{2}$, respectively. Suppose that $A : H_{1} \rightarrow H_{2}$ is a nonzero bounded linear operator, $f : C\rightarrow H_{1}$ is a monotone and *k*-Lipschitz continuous mapping, and $g: H_{2} \rightarrow H_{2}$ is a *δ*-inverse strongly monotone mapping. Suppose that $T:H_{2} \rightarrow H_{2}$ and $S : C \rightarrow C$ are nonexpansive. Let $\{\mu_{n}\}, \{ \alpha_{n}\} \subset(0,1)$, $\{\gamma_{n}\} \subset[a, b]$ for some $a, b \in(0, \frac{1}{\|A\|^{2}})$ and $\{\lambda_{n}\} \subset[c, d]$ for some $c, d \in(0,\frac{1}{k})$.

Firstly, we present an algorithm for solving the variational inequality problems and split common fixed point problems, that is, finding a point $x^{*}$ such that

3.1 $$ x^{*} \in\operatorname{VI}(C, f) \cap\operatorname{Fix}(S) \quad \text{and}\quad Ax^{*} \in\operatorname{Fix}(T). $$

### Theorem 3.1

*Set* $\varGamma= \{z \in\operatorname{VI}(C, f)\cap \operatorname{Fix}(S) : Az \in\operatorname{Fix}(T)\}$ *and assume that* $\varGamma\neq\emptyset$. *Let the sequences* $\{x_{n}\}$, $\{y_{n}\}$ *and* $\{z_{n}\}$ *be generated by* $x_{1} = x \in C$ *and*

3.2 $$\begin{aligned}& y_{n} = \mu_{n} x_{n}+(1-\mu_{n})P_{C} \bigl(x_{n} - \gamma_{n} A^{*}(I - T)Ax_{n}\bigr), \\& z_{n} = P_{C}\bigl(y_{n} - \lambda_{n}f(y_{n}) \bigr), \\& x_{n+1} = \alpha_{n} y_{n} + (1- \alpha_{n}) SP_{C}\bigl(y_{n} - \lambda _{n}f(z_{n})\bigr), \end{aligned}$$

*for each* $n \in {\mathbb {N}}$. *Then the sequence* $\{x_{n}\}$ *converges weakly to a point* $z \in\varGamma$, *where* $z = \lim_{n \rightarrow \infty} P_{\varGamma}x_{n}$.

### Proof

It follows from Theorem 3.1 [32] that $P_{C}(I-\gamma _{n}A^{*}(I-T)A)$ is $\frac{1+\gamma_{n}\|A\|^{2}}{2}$-averaged. It is easy to see from Lemma 2.2 [25] that $\mu_{n} I+(1-\mu_{n})P_{C}(I - \gamma_{n} A^{*}(I - T)A)$ is $\mu_{n}+(1-\mu_{n})\frac {1+\gamma_{n}\|A\|^{2}}{2}$-averaged. So, $y_{n}$ can be rewritten as

3.3 $$ y_{n}=(1-\beta_{n})x_{n}+\beta_{n}V_{n}x_{n}, $$

where $\beta_{n}=\mu_{n}+(1-\mu_{n})\frac{1+\gamma_{n}\|A\|^{2}}{2}$ and $V_{n}$ is a nonexpansive mapping for each $n \in {\mathbb {N}}$.

Let $u \in\varGamma$, we get that

3.4 $$\begin{aligned} \|y_{n}-u\|^{2} =& \bigl\Vert (1-\beta_{n}) (x_{n}-u)+\beta_{n}(V_{n}x_{n}-u) \bigr\Vert ^{2} \\ =& (1-\beta_{n})\|x_{n}-u\|^{2}+ \beta_{n}\|V_{n}x_{n}-u\|^{2} \\ &{} -\beta_{n}(1-\beta_{n})\|x_{n}-V_{n}x_{n} \|^{2} \\ \leq& \|x_{n}-u\|^{2}-\beta_{n}(1- \beta_{n})\|x_{n}-V_{n}x_{n} \|^{2} \\ \leq& \|x_{n} -u\|^{2}. \end{aligned}$$

Thus

3.5 $$ \beta_{n}(1-\beta_{n})\|x_{n}-V_{n}x_{n} \|^{2} \leq\|x_{n}-u\|^{2}-\|y_{n}-u \|^{2}. $$

Set $t_{n}=P_{C}(y_{n}-\lambda_{n} fz_{n})$ for all $n \geq0$. It follows from Lemma 2.1 that

$$\begin{aligned} \Vert t_{n}-u \Vert ^{2} \leq& \bigl\Vert y_{n}-\lambda_{n} f(z_{n})-u \bigr\Vert ^{2}- \bigl\Vert y_{n}-\lambda _{n}f(z_{n})-t_{n} \bigr\Vert ^{2} \\ \leq& \Vert y_{n}-u \Vert ^{2}- \Vert y_{n}-t_{n} \Vert ^{2}+2\lambda_{n} \bigl\langle f(z_{n}), u-t_{n}\bigr\rangle \\ =& \Vert y_{n}-u \Vert ^{2}- \Vert y_{n}-t_{n} \Vert ^{2}+2\lambda_{n} \bigl(\bigl\langle f(z_{n})-f(u), u-z_{n}\bigr\rangle \\ &{} +\bigl\langle f(u), u-z_{n}\bigr\rangle +\bigl\langle f(z_{n}), z_{n}-t_{n}\bigr\rangle \bigr) \\ \leq& \Vert y_{n}-u \Vert ^{2}- \Vert y_{n}-t_{n} \Vert ^{2}+2\lambda_{n} \bigl\langle f(z_{n}), z_{n}-t_{n}\bigr\rangle \\ =& \Vert y_{n}-u \Vert ^{2}- \Vert y_{n}-z_{n} \Vert ^{2}-2\langle y_{n}-z_{n}, z_{n}-t_{n}\rangle- \Vert z_{n}-t_{n} \Vert ^{2} \\ &{}+2\lambda_{n}\bigl\langle f(z_{n}), z_{n}-t_{n} \bigr\rangle \\ =& \Vert y_{n}-u \Vert ^{2}- \Vert y_{n}-z_{n} \Vert ^{2}- \Vert z_{n}-t_{n} \Vert ^{2} \\ &{} +2\bigl\langle y_{n}-\lambda_{n}f(z_{n})-z_{n}, t_{n}-z_{n}\bigr\rangle . \end{aligned}$$

Using Lemma 2.1 again, this yields

$$\begin{aligned} \bigl\langle y_{n}-\lambda_{n}f(z_{n})-z_{n}, t_{n}-z_{n}\bigr\rangle =& \bigl\langle y_{n}- \lambda_{n}f(y_{n})-z_{n}, t_{n}-z_{n} \bigr\rangle \\ &{} +\bigl\langle \lambda_{n}f(y_{n})-\lambda_{n}f(z_{n}), t_{n}-z_{n}\bigr\rangle \\ \leq& \bigl\langle \lambda_{n}f(y_{n})-\lambda_{n}f(z_{n}), t_{n}-z_{n}\bigr\rangle \\ \leq& \lambda_{n}k\|y_{n}-z_{n}\| \|t_{n}-z_{n}\|, \end{aligned}$$

and so

$$ \|t_{n}-u\|^{2} \leq\|y_{n}-u\|^{2}- \|y_{n}-z_{n}\|^{2}-\|z_{n}-t_{n} \|^{2}+2\lambda_{n}k\| y_{n}-z_{n}\| \|t_{n}-z_{n}\|. $$

For each $n \in {\mathbb {N}}$, we obtain that

$$\begin{aligned} 0 \leq& \bigl( \Vert t_{n}-z_{n} \Vert - \lambda_{n}k\|y_{n}-z_{n}\|\bigr)^{2} \\ =&\|t_{n}-z_{n}\|^{2}-2\lambda_{n}k \|t_{n}-z_{n}\|\|y_{n}-z_{n}\|+ \lambda_{n}^{2}k^{2}\| y_{n}-z_{n} \|^{2}. \end{aligned}$$

That is,

$$ 2\lambda_{n}k\|t_{n}-z_{n}\|\|y_{n}-z_{n} \| \leq\|t_{n}-z_{n}\|^{2} + \lambda _{n}^{2}k^{2}\|y_{n}-z_{n} \|^{2}. $$

So,

3.6 $$\begin{aligned} \|t_{n}-u\|^{2} \leq& \|y_{n}-u\|^{2}- \|y_{n}-z_{n}\|^{2}-\|z_{n}-t_{n} \|^{2}+\|t_{n}-z_{n}\| ^{2} \\ &{}+ \lambda_{n}^{2}k^{2}\|y_{n}-z_{n} \|^{2} \\ =& \|y_{n}-u\|^{2}+ \bigl(\lambda_{n}^{2}k^{2}-1 \bigr)\|y_{n}-z_{n}\|^{2} \\ \leq& \|y_{n}-u\|^{2}. \end{aligned}$$

By the convexity of the norm and (3.6), we have

3.7 $$\begin{aligned} \Vert x_{n+1}-u \Vert ^{2} =& \bigl\Vert \alpha_{n}y_{n}+(1-\alpha_{n})S(t_{n})-u \bigr\Vert ^{2} \\ =& \bigl\Vert \alpha_{n}(y_{n}-u)+(1- \alpha_{n}) \bigl(S(t_{n})-u\bigr) \bigr\Vert ^{2} \\ =& \alpha_{n} \Vert y_{n}-u \Vert ^{2}+(1- \alpha_{n}) \bigl\Vert S(t_{n})-u \bigr\Vert ^{2} \\ &{}-\alpha_{n}(1-\alpha_{n}) \bigl\Vert y_{n}-u-\bigl(S(t_{n})-u\bigr) \bigr\Vert ^{2} \\ \leq& \alpha_{n} \Vert y_{n}-u \Vert ^{2}+(1- \alpha_{n}) \bigl\Vert S(t_{n})-S(u) \bigr\Vert ^{2} \\ \leq& \alpha_{n} \Vert y_{n}-u \Vert ^{2}+(1- \alpha_{n}) \Vert t_{n}-u \Vert ^{2} \\ \leq& \alpha_{n} \Vert y_{n}-u \Vert ^{2}+(1- \alpha_{n})\bigl[ \Vert y_{n}-u \Vert ^{2}+ \bigl(\lambda_{n}^{2}k^{2}-1\bigr) \Vert y_{n}-z_{n} \Vert ^{2}\bigr] \\ =& \Vert y_{n}-u \Vert ^{2}+(1-\alpha_{n}) \bigl(\lambda_{n}^{2}k^{2}-1\bigr) \Vert y_{n}-z_{n} \Vert ^{2} \\ \leq& \Vert y_{n}-u \Vert ^{2} \leq \Vert x_{n}-u \Vert ^{2}. \end{aligned}$$

Hence, there exists $c\geq0$ such that

3.8 $$ \lim_{n \rightarrow \infty}\|x_{n}-u\|=c, $$

and then $\{x_{n}\}$ is bounded. This implies that $\{y_{n}\}$ and $\{t_{n}\} $ are also bounded. From (3.5) and (3.7), we deduce that

$$ \beta_{n}(1-\beta_{n})\|x_{n}-V_{n}x_{n} \|^{2} \leq\|x_{n}-u\|^{2}-\|x_{n+1}-u \|^{2}. $$

Therefore, it follows from (3.8) that

$$x_{n}-V_{n}x_{n} \rightarrow 0, \quad \text{as } n\rightarrow \infty. $$

By (3.3), we get that

3.9 $$ x_{n}-y_{n} = \beta_{n}(x_{n}-V_{n}x_{n}) \rightarrow 0, \quad \text{as } n\rightarrow \infty. $$

Relation (3.7) implies

$$ (1-\alpha_{n}) \bigl(1-\lambda_{n}^{2}k^{2} \bigr)\|y_{n}-z_{n}\|^{2} \leq \|y_{n}-u \|^{2}-\| x_{n+1}-u\|^{2}, $$

and so

3.10 $$ y_{n}-z_{n} \rightarrow 0, \quad \text{as } n\rightarrow \infty. $$

Moreover, by the definition of $z_{n}$, we have

$$\begin{aligned} \Vert z_{n}-t_{n} \Vert ^{2} =& \bigl\Vert P_{C}\bigl(y_{n}-\lambda_{n}f(y_{n}) \bigr)-P_{C}\bigl(y_{n}-\lambda _{n}f(z_{n}) \bigr) \bigr\Vert ^{2} \\ \leq& \bigl\Vert \bigl(y_{n}-\lambda_{n}f(y_{n}) \bigr)-\bigl(y_{n}-\lambda_{n}f(z_{n})\bigr) \bigr\Vert ^{2} \\ =& \bigl\Vert \lambda_{n}f(z_{n})-\lambda_{n}f(y_{n}) \bigr\Vert ^{2} \\ \leq& \lambda_{n}^{2}k^{2} \Vert z_{n}-y_{n} \Vert ^{2}. \end{aligned}$$

Hence

3.11 $$ z_{n} - t_{n} \rightarrow 0, \quad \text{as } n \rightarrow \infty. $$

Using the triangle inequality, we see that

$$ \|y_{n}-t_{n}\| \leq\|y_{n}-z_{n}\| + \|z_{n}-t_{n}\| $$

and

$$ \|x_{n}-z_{n}\| \leq\|x_{n}-y_{n}\|+ \|y_{n}-z_{n}\|. $$

This implies that

3.12 $$ y_{n}-t_{n} \rightarrow 0 \quad \text{and}\quad x_{n}-z_{n} \rightarrow 0,\quad \text{as } n \rightarrow \infty. $$

The definition of $y_{n}$ implies

$$ (1-\mu_{n}) \bigl(x_{n}-P_{C}\bigl(x_{n}- \gamma_{n}A^{*}(I-T)Ax_{n}\bigr)\bigr)=x_{n}-y_{n}. $$

Thus

3.13 $$ x_{n}-P_{C}\bigl(x_{n}-\gamma_{n}A^{*}(I-T)Ax_{n} \bigr) \rightarrow 0, \quad \text{as } n \rightarrow \infty . $$

Let $z \in\omega_{w}(x_{n})$. Then there exists a subsequence $\{x_{n_{i}}\} $ of $\{x_{n}\}$ which converges weakly to *z*. We obtain that $\{ A^{*}(I-T)Ax_{n_{i}}\}$ is bounded because $A^{*}(I-T)A$ is $\frac{1}{2\|A\| ^{2}}$-inverse strongly monotone. By the firm nonexpansiveness of $P_{C}$, we see that

$$\begin{aligned}& \bigl\Vert P_{C}\bigl(I-\gamma_{n_{i}}A^{*}(I-T)A \bigr)x_{n_{i}}-P_{C}\bigl(I-\hat{\gamma }A^{*}(I-T)A \bigr)x_{n_{i}} \bigr\Vert \\& \quad \leq |\gamma_{n_{i}}-\hat{\gamma}| \bigl\Vert A^{*}(I-T)Ax_{n_{i}} \bigr\Vert . \end{aligned}$$

Without loss of generality, we may assume that $\gamma_{n_{i}} \rightarrow \hat {\gamma} \in(0,\frac{1}{\|A\|^{2}})$, and so

3.14 $$ P_{C}\bigl(I-\gamma_{n_{i}}A^{*}(I-T)A\bigr)x_{n_{i}}-P_{C} \bigl(I-\hat{\gamma }A^{*}(I-T)A\bigr)x_{n_{i}} \rightarrow 0,\quad i \rightarrow \infty. $$

From (3.13), (3.14) and

$$\begin{aligned}& \bigl\Vert x_{n_{i}}-P_{C}\bigl(I-\hat{\gamma}A^{*}(I-T)A \bigr)x_{n_{i}} \bigr\Vert \\& \quad \leq \bigl\Vert x_{n_{i}}- P_{C}\bigl(I- \gamma_{n_{i}}A^{*}(I-T)A\bigr)x_{n_{i}} \bigr\Vert \\& \qquad {}+ \bigl\Vert P_{C}\bigl(I-\gamma_{n_{i}}A^{*}(I-T)A \bigr)x_{n_{i}}-P_{C}\bigl(I-\hat {\gamma}A^{*}(I-T)A \bigr)x_{n_{i}} \bigr\Vert , \end{aligned}$$

we have

3.15 $$ x_{n_{i}}-P_{C}\bigl(I-\hat{\gamma}A^{*}(I-T)A \bigr)x_{n_{i}} \rightarrow 0, \quad \text{as } i \rightarrow \infty. $$

By the demiclosedness principle [33], we have

$$z \in\operatorname{Fix}\bigl(P_{C}\bigl(I-\hat{\gamma}A^{*}(I-T)A\bigr) \bigr). $$

Using Corollary 2.9 [32], this yields

3.16 $$ z \in C \cap A^{-1}\operatorname{Fix}(T). $$

Next, we claim that *z*∈ $\operatorname{VI}(C,f)$. From (3.9), (3.10) and (3.11), we know that $y_{n_{i}} \rightharpoonup z$, $z_{n_{i}} \rightharpoonup z$ and $t_{n_{i}} \rightharpoonup z$. Define the set-valued mapping $B: H \rightrightarrows H$ by

$$ Bv = \textstyle\begin{cases} f(v) + N_{C}v, &\text{if } \forall v\in C; \\ \emptyset, &\text{if } \forall v\notin C. \end{cases} $$

In [27], this implies that *B* is maximal monotone and we have $0 \in Bv$ iff $v \in\operatorname{VI}(C,f)$. If $(v,w) \in D(B)$, then $w \in Bv=f(v)+N_{C}v$ and so $w-f(v) \in N_{C}v$. Thus, for any $p \in C$, we get

3.17 $$ \bigl\langle v-p, w-f(v)\bigr\rangle \geq0. $$

Since $v \in C$, it follows from the definition of $z_{n}$ and Lemma 2.1 that

$$ \langle y_{n} - \lambda_{n}fy_{n}-z_{n},z_{n}-v \rangle\geq0. $$

Consequently,

$$ \biggl\langle \frac{z_{n}-y_{n}}{\lambda_{n}}+f(y_{n}), v-z_{n}\biggr\rangle \geq0. $$

By using (3.17) with $\{z_{n_{i}}\}$, we obtain

$$ \bigl\langle w -f(v), v -z_{n_{i}}\bigr\rangle \geq0. $$

Thus

$$\begin{aligned} \langle w,v-z_{n_{i}}\rangle \geq& \bigl\langle f(v),v-z_{n_{i}} \bigr\rangle \\ \geq& \bigl\langle f(v),v-z_{n_{i}}\bigr\rangle - \biggl\langle \frac {z_{n_{i}}-y_{n_{i}}}{\lambda_{n_{i}}}+ f(y_{n_{i}}),v-z_{n_{i}}\biggr\rangle \\ =& \bigl\langle f(v)-f(z_{n_{i}}),v-z_{n_{i}}\bigr\rangle + \bigl\langle f(z_{n_{i}}) - f(y_{n_{i}}) ,v-z_{n_{i}}\bigr\rangle \\ &{}-\biggl\langle \frac{z_{n_{i}}-y_{n_{i}}}{\lambda_{n_{i}}},v-z_{n_{i}}\biggr\rangle \\ \geq& \bigl\langle f(z_{n_{i}}) - f(y_{n_{i}}) ,v-z_{n_{i}}\bigr\rangle -\biggl\langle \frac{z_{n_{i}}-y_{n_{i}}}{\lambda_{n_{i}}},v-z_{n_{i}} \biggr\rangle . \end{aligned}$$

By taking $i\rightarrow \infty$ in the above inequality, we deduce

$$ \langle w,v-z\rangle\geq0. $$

By the maximal monotonicity of *B*, we get $0 \in Bz$ and so $z \in \operatorname{VI}(C,f)$. Now, we will show that $z \in\operatorname{Fix}(S)$. Since *S* is nonexpansive, it follows from (3.4) and (3.6) that

$$ \bigl\Vert S(t_{n})-u \bigr\Vert = \bigl\Vert S(t_{n})-S(u) \bigr\Vert \leq \Vert t_{n}-u \Vert \leq \Vert y_{n}-u \Vert \leq \Vert x_{n}-u \Vert , $$

and by taking limit superior in the above inequalities and using (3.8), we obtain

$$ \limsup_{n \rightarrow \infty} \bigl\| S(t_{n})-u\bigr\| \leq c \quad \text{and} \quad \limsup_{n \rightarrow \infty} \|y_{n} -u \| \leq c. $$

Further,

$$\begin{aligned} \lim_{n \rightarrow \infty} \bigl\Vert \alpha_{n}(y_{n}-u)+(1- \alpha _{n}) \bigl(S(t_{n})-u\bigr) \bigr\Vert =& \lim _{n \rightarrow \infty} \bigl\Vert \alpha _{n}y_{n}+(1- \alpha_{n})S(t_{n})-u \bigr\Vert \\ =& \lim_{n \rightarrow \infty} \Vert x_{n+1}-u \Vert \\ =& c, \end{aligned}$$

and so Lemma 2.5 implies

3.18 $$ \lim_{n \rightarrow \infty} \bigl\| S(t_{n})-y_{n}\bigr\| =0. $$

From the fact that

$$\begin{aligned} \bigl\Vert S(y_{n})-y_{n} \bigr\Vert =& \bigl\Vert S(y_{n})-S(t_{n})+S(t_{n})-y_{n} \bigr\Vert \\ \leq& \bigl\Vert S(y_{n})-S(t_{n}) \bigr\Vert + \bigl\Vert S(t_{n})-y_{n} \bigr\Vert \\ \leq& \Vert y_{n}-t_{n} \Vert + \bigl\Vert S(t_{n})-y_{n} \bigr\Vert , \end{aligned}$$

(3.12) and (3.18), we have

$$ \lim_{n \rightarrow \infty} \bigl\Vert S(y_{n})-y_{n} \bigr\Vert =0. $$

This implies that

$$ \lim_{i \rightarrow \infty} \bigl\Vert (I-S) (y_{n_{i}}) \bigr\Vert = \lim_{i \rightarrow \infty} \bigl\Vert y_{n_{i}}-S(y_{n_{i}}) \bigr\Vert =0. $$

Now, by the demiclosedness principle [33], we have $z \in\operatorname{Fix}(S)$. Consequently, $\omega_{w}(x_{n}) \subset\varGamma$. By Lemma 2.6, the sequence $\{x_{n}\}$ is weakly convergent to a point *z* in *Γ* and Lemma 3.2 [28] assures $z=\lim_{n \rightarrow \infty} P_{\varGamma}x_{n}$. □

### Remark 3.2

We can obtain the following statements:

(i) If $f=0$, $T=P_{Q}$, and $S=I$, then problem (3.1) coincides with the SFP and algorithm (3.2) reduces to algorithm (1.8) for solving the SFP.
(ii) If $T=I$, then problem (3.1) coincides with the VIP and FPP and algorithm (3.2) reduces to algorithm (1.5) for solving the VIP and FPP.
(iii) If $S=I$, then problem (3.1) coincides with problem 3.1 in [32] and if $\alpha_{n}, \mu_{n} =0$, we obtain that algorithm (3.2) reduces to algorithm 3.2 in [32].

The following result provides suitable conditions in order to guarantee the existence of a common solution of the split variational inequality problems and fixed point problems, that is, finding a point $x^{*}$ such that

3.19 $$ x^{*}\in\operatorname{VI}(C,f) \cap\operatorname{Fix}(S)\quad \text{and}\quad Ax^{*} \in\operatorname{VI}(Q,g). $$

### Theorem 3.3

*Set* $\varGamma= \{z \in\operatorname{VI}(C,f) \cap\operatorname{Fix}(S) : Az \in\operatorname{VI}(Q,g)\}$ *and assume that* $\varGamma \neq\emptyset$. *Let the sequences* $\{x_{n}\}$, $\{y_{n}\}$ *and* $\{z_{n}\}$ *be generated by* $x_{1} = x \in C$ *and*

3.20 $$\begin{aligned}& y_{n} = \mu_{n} x_{n}+(1-\mu_{n})P_{C} \bigl(x_{n} - \gamma_{n} A^{*}\bigl(I - P_{Q}(I-\theta g)\bigr)Ax_{n}\bigr), \\& z_{n} = P_{C}\bigl(y_{n} - \lambda_{n}f(y_{n}) \bigr), \\& x_{n+1} = \alpha_{n} y_{n} + (1- \alpha_{n}) SP_{C}\bigl(y_{n} - \lambda _{n}f(z_{n})\bigr), \end{aligned}$$

*for each* $n \in N$, *where* $\theta\in(0,2\delta)$. *Then the sequence* $\{x_{n}\}$ *converges weakly to a point* $z \in\varGamma$, *where* $z = \lim_{n \rightarrow \infty} P_{\varGamma}x_{n}$.

### Proof

It is clear from *δ*-inverse strongly monotonicity of *g* that it is $\frac{1}{\delta}$-Lipschitz continuous and so, for $\theta\in (0,2\delta)$, we obtain that $I-\theta g$ is nonexpansive. Since $P_{Q}$ is firmly nonexpansive, then $P_{Q}(I-\theta g)$ is nonexpansive. By taking $T=P_{Q}(I-\theta g)$ in Theorem 3.1, we obtain that $\{ x_{n}\}$ converges weakly to a point $z\in\operatorname{VI}(C,f) \cap\operatorname {Fix}(S)$ and $Az \in\operatorname{Fix}(P_{Q}(I-\theta g))$. It follows from $Az =P_{Q}(I-\theta g)Az$ and Lemma 2.1 that $Az \in\operatorname {VI}(Q,g)$. This completes the proof. □

### Remark 3.4

We can obtain the following statements:

(i) If $f=0$, $g=0$, and $S=I$, then problem (3.19) coincides with the SFP and algorithm (3.20) reduces to algorithm (1.8) for solving the SFP.
(ii) If $g=0$ and $Q=H_{2}$, then problem (3.19) coincides with the VIP and FPP and algorithm (3.20) reduces to algorithm (1.5) for solving the VIP and FPP.
(iii) If $S=I$, then problem (3.19) coincides with problem 3.1 in [32] and if $\alpha_{n}, \mu_{n} =0$, then algorithm (3.20) reduces to algorithm (1.10).

## Applications

In this section, by using the main results, we give some applications to the weak convergence of the produced algorithms for the equilibrium problem, zero point problem and convex minimization problem.

The equilibrium problem was formulated by Blum and Oettli [4] in 1994 for finding a point $x^{*}$ such that

4.1 $$ F\bigl(x^{*},y\bigr) \geq0 \quad \text{for all } y \in C, $$

where $F:C \times C \rightarrow {\mathbb {R}}$ is a bifunction. The solution set of equilibrium problem (4.1) is denoted by $\operatorname{EP}(C,F)$.

In [4], we know that if *F* is a bifunction such that

1. $F(x,x)=0$ for all $x \in C$;
2. *F* is monotone, that is, $F(x,y) + F(y,x) \leq0$ for all $x, y \in C$;
3. for each $x, y, z \in C$, $\limsup_{t\downarrow0} F(tz + (1 - t)x, y) \leq F(x, y)$;
4. for each fixed $x \in C$, $y \mapsto F(x,y)$ is lower semicontinuous and convex,

then there exists $z \in C$ such that

$$ F(z,y) + \frac{1}{r} \langle y - z, z - x\rangle\geq0,\quad \forall y \in C, $$

where *r* is a positive real number and $x \in H$.

For $r > 0$ and $x\in H$, the resolvent $T_{r} : H \rightarrow C$ of a bifunction *F* which satisfies conditions (A1)–(A4) is formulated as follows:

$$ T_{r}x=\biggl\{ z \in C : F(z,y) +\frac{1}{r} \langle y-z, z-x \rangle\geq0, \text{for all } y \in C\biggr\} \quad \text{for all } x \in H, $$

and has the following properties:

(i) $T_{r}$ is single-valued and firmly nonexpansive;
(ii) $\operatorname{Fix}(T_{r}) = \operatorname{EP}(C,F)$;
(iii) $\operatorname{EP}(C,F)$ is closed and convex.

For more details, see [13].

The following result is related to the equilibrium problems by applying Theorem 3.1.

### Theorem 4.1

*Let* $F : C \times C \rightarrow {\mathbb {R}}$ *be a bifunction satisfying conditions* (A1)*–*(A4). *Set* $\varGamma= \{z \in\operatorname{VI}(C, f)\cap \operatorname {Fix}(S) : Az \in\operatorname{EP}(C,F)\}$ *and suppose that* $\varGamma\neq \emptyset$. *Let the sequences* $\{x_{n}\}$, $\{y_{n}\}$ *and* $\{z_{n}\}$ *be generated by* $x_{1} = x \in C$ *and*

4.2 $$ \textstyle\begin{cases} y_{n} = \mu_{n} x_{n}+(1-\mu_{n})P_{C}(x_{n} - \gamma_{n} A^{*}(I - T_{r})Ax_{n}), \\ z_{n} = P_{C}(y_{n} - \lambda_{n}f(y_{n})), \\ x_{n+1} = \alpha_{n} y_{n} + (1-\alpha_{n}) SP_{C}(y_{n} - \lambda_{n}f(z_{n})), \end{cases} $$

*for each* $n \in {\mathbb {N}}$, *where* $T_{r}$ *is a resolvent of* *F* *for* $r > 0$. *Then the sequence* $\{x_{n}\}$ *converges weakly to a point* $z \in\varGamma $, *where* $z = \lim_{n \rightarrow \infty} P_{\varGamma}x_{n}$.

### Proof

Since $T_{r}$ is nonexpansive, the proof follows from Theorem 3.1 by taking $T_{r}=T$. □

The following results are the application of Theorem 3.1 to the zero point problem.

### Theorem 4.2

*Let* $B : H_{2} \rightrightarrows{H_{2}}$ *be a maximal monotone mapping with* $D(B) \neq\emptyset$. *Set* $\varGamma= \{z \in\operatorname{VI}(C, f)\cap \operatorname{Fix}(S) : Az \in B^{-1}0\}$ *and assume that* $\varGamma\neq \emptyset$. *Let the sequences* $\{x_{n}\}$, $\{y_{n}\}$ *and* $\{z_{n}\}$ *be generated by* $x_{1} = x \in C$ *and*

4.3 $$ \textstyle\begin{cases} y_{n} = \mu_{n} x_{n}+(1-\mu_{n})P_{C}(x_{n} - \gamma_{n} A^{*}(I - J_{r})Ax_{n}), \\ z_{n} = P_{C}(y_{n} - \lambda_{n}f(y_{n})), \\ x_{n+1} = \alpha_{n} y_{n} + (1-\alpha_{n}) SP_{C}(y_{n} - \lambda_{n}f(z_{n})), \end{cases} $$

*for each* $n \in {\mathbb {N}}$, *where* $J_{r}$ *is a resolvent of* *B* *for* $r > 0$. *Then the sequence* $\{x_{n}\}$ *converges weakly to a point* $z \in\varGamma $, *where* $z = \lim_{n \rightarrow \infty} P_{\varGamma}x_{n}$.

### Proof

Since $J_{r}$ is firmly nonexpansive and $\operatorname{Fix}(J_{r})=B^{-1}0$, the proof follows from Theorem 3.1 by taking $J_{r}=T$. □

### Theorem 4.3

*Let* $B : H_{2}\rightrightarrows{H_{2}}$ *be a maximal monotone mapping with* $D(B) \neq\emptyset$ *and* $F : H_{2} \rightarrow H_{2}$ *be a* *δ*-*inverse strongly monotone mapping*. *Set* $\varGamma= \{z \in\operatorname{VI}(C,f) \cap\operatorname{Fix}(S) : Az \in(B + F)^{-1}0\}$ *and assume that* $\varGamma\neq\emptyset$. *Let the sequences* $\{x_{n}\}$, $\{y_{n}\}$ *and* $\{z_{n}\}$ *be generated by* $x_{1} = x \in C$ *and*

4.4 $$ \textstyle\begin{cases} y_{n} = \mu_{n} x_{n}+(1-\mu_{n})P_{C}(x_{n} - \gamma_{n} A^{*}(I - J_{r}(I-rF))Ax_{n}), \\ z_{n} = P_{C}(y_{n} - \lambda_{n}f(y_{n})), \\ x_{n+1} = \alpha_{n} y_{n} + (1-\alpha_{n}) SP_{C}(y_{n} - \lambda_{n}f(z_{n})), \end{cases} $$

*for each* $n \in {\mathbb {N}}$, *where* $J_{r}$ *is a resolvent of* *B* *for* $r \in (0,2\delta)$. *Then the sequence* $\{x_{n}\}$ *converges weakly to a point* $z \in\varGamma$, *where* $z = \lim_{n \rightarrow \infty} P_{\varGamma}x_{n}$.

### Proof

Since *F* is *δ*-inverse strongly monotone, then $I-rF$ is nonexpansive. By the nonexpansiveness of $J_{r}$, we obtain that $J_{r}(I-rF)$ is also nonexpansive. We know that $z \in(B+F)^{-1}0$ if and only if $z=J_{r}(I-rF)z$. Thus the proof follows from Theorem 3.1 by taking $J_{r}(I-rF)=T$. □

Let *ϕ* be a real-valued convex function from *C* to ${\mathbb {R}}$, the typical form of constrained convex minimization problem is finding a point $x^{*} \in C$ satisfying

4.5 $$ \phi\bigl(x^{*}\bigr)=\min_{x \in C} \phi(x). $$

Denote the solution set of constrained convex minimization problem (4.5) by $\arg\min_{x \in C} \phi(x)$.

By applying Theorem 3.3, we get the following result.

### Theorem 4.4

*Let* $\phi: H_{2} \rightarrow {\mathbb {R}}$ *be a differentiable convex function and suppose that* ∇*ϕ* *is a* *δ*-*inverse strongly monotone mapping*. *Set* $\varGamma= \{z \in\operatorname{VI}(C,f) \cap\operatorname{Fix}(S) : Az \in\arg \min_{y\in Q} \phi(y)\}$ *and assume that* $\varGamma\neq\emptyset$. *Let the sequences* $\{x_{n}\}$, $\{y_{n}\}$ *and* $\{z_{n}\}$ *be generated by* $x_{1} = x \in C$ *and*

4.6 $$ \textstyle\begin{cases} y_{n} = \mu_{n} x_{n}+(1-\mu_{n})P_{C}(x_{n} - \gamma_{n} A^{*}(I - P_{Q}(I-\theta \nabla\phi))Ax_{n}), \\ z_{n} = P_{C}(y_{n} - \lambda_{n}f(y_{n})), \\ x_{n+1} = \alpha_{n} y_{n} + (1-\alpha_{n}) SP_{C}(y_{n} - \lambda_{n}f(z_{n})), \end{cases} $$

*for each* $n \in {\mathbb {N}}$, *where* $\theta\in(0,2\delta)$. *Then the sequence* $\{x_{n}\}$ *converges weakly to a point* $z\in\varGamma$, *where* $z = \lim_{n \rightarrow \infty} P_{\varGamma}x_{n}$.

### Proof

Since *ϕ* is convex, for each $x,y \in C$, we have

$$ \phi\bigl(x+\lambda(z-x)\bigr) \leq(1-\lambda)\phi(x)+\lambda\phi(z)\quad \text{for all } \lambda\in(0,1). $$

It follows that $\langle\nabla\phi(x),x-z\rangle\geq\phi(x) -\phi (z) \geq\langle\nabla\phi(z),x-z\rangle$. This implies that ∇*ϕ* is monotone. By Lemma 4.6 [32] and taking $g= \nabla\phi$, the proof follows from Theorem 3.3. □

We obtain the following result for solving the split minimization problems and fixed point problems by applying Theorem 3.3.

### Theorem 4.5

*Let* $\phi_{1}: H_{1} \rightarrow {\mathbb {R}}$ *and* $\phi_{2} : H_{2} \rightarrow {\mathbb {R}}$ *be differentiable convex functions*. *Suppose that* $\nabla\phi_{1}$ *is a* *k*-*Lipschitz continuous mapping and* $\nabla\phi_{2}$ *is* *δ*-*inverse strongly monotone*. *Set* $\varGamma= \{z \in\operatorname{arg\,min}_{x\in C} \phi_{1}(x) \cap\operatorname {Fix}(S) : Az \in\operatorname{arg\,min}_{y\in Q} \phi_{2}(y)\}$ *and assume that* $\varGamma\neq\emptyset$. *Let the sequences* $\{x_{n}\}$, $\{y_{n}\}$ *and* $\{z_{n}\}$ *be generated by* $x_{1} = x \in C$ *and*

4.7 $$ \textstyle\begin{cases} y_{n} = \mu_{n} x_{n}+(1-\mu_{n})P_{C}(x_{n} - \gamma_{n} A^{*}(I - P_{Q}(I-\theta \nabla\phi_{2}))Ax_{n}), \\ z_{n} = P_{C}(y_{n} - \lambda_{n}\nabla\phi_{1}(y_{n})), \\ x_{n+1} = \alpha_{n} y_{n} + (1-\alpha_{n}) SP_{C}(y_{n} - \lambda_{n}\nabla \phi_{1}(z_{n})), \end{cases} $$

*for each* $n \in {\mathbb {N}}$, *where* $\theta\in(0,2\delta)$. *Then the sequence* $\{x_{n}\}$ *converges weakly to a point* $z \in\varGamma$, *where* $z = \lim_{n \rightarrow \infty} P_{\varGamma}x_{n}$.

### Proof

The convexity of $\phi_{1}$ implies that $\nabla\phi_{1}$ is monotone. The result follows from Lemma 4.6 [32] by taking $f = \nabla \phi_{1}$ and $g = \nabla\phi_{2}$ in Theorem 3.3. □
